# Dual pH- and Thermo-Sensitive Poly(N-isopropylacrylamide-co-allylamine) Nanogels for Curcumin Delivery: Swelling–Deswelling Behavior and Phase Transition Mechanism

**DOI:** 10.3390/gels9070536

**Published:** 2023-07-01

**Authors:** Madhappan Santhamoorthy, Seong-Cheol Kim

**Affiliations:** School of Chemical Engineering, Yeungnam University, Gyeongsan 38541, Republic of Korea

**Keywords:** pNIPAm copolymer, dual stimuli, drug delivery, phase transition, cytocompatibility

## Abstract

Curcumin (Cur) is a beneficial ingredient with numerous bioactivities. However, due to its low solubility and poor bioavailability, its therapeutic application is limited. In this work, we prepared poly-N-isopropylacrylamide p(NIPAm) and polyallylamine p(Am)-based nanogel (p(NIPAm-co-Am)) NG for a dual pH- and temperature-sensitive copolymer system for drug delivery application. In this copolymer system, the p(NIPAm) segment was incorporated to introduce thermoresponsive behavior and the p(Am) segment was incorporated to introduce drug binding sites (amine groups) in the resulting (p(NIPAm-co-Am)) NG system. Various instrumental characterizations including ^1^H nuclear magnetic resonance (^1^H NMR) spectroscopy, Fourier transform infrared (FT-IR) analysis, scanning electron microscopy (SEM), zeta potential, and particle size analysis were performed to confirm the copolymer synthesis. Curcumin (Cur), an anticancer bioactive substance, was employed to assess the in vitro drug loading and release performance of the resulting copolymer nanogels system at varied pH levels (pH 7.2, 6.5, and 4.0) and temperatures (25 °C, 37 °C, and 42 °C). The cytocompatibility of the p(NIPAm-co-Am) NG sample was also tested on MDA-MB-231 cells at various sample concentrations. All the study results indicate that the p(NIPAm-co-Am) NG produced might be effective for drug loading and release under pH and temperature dual-stimuli conditions. As a result, the p(NIPAm-co-Am) NG system has the potential to be beneficial in the use of drug delivery applications in cancer therapy.

## 1. Introduction

Curcumin (Cur) is derived from the spice turmeric and has been shown to have a wide variety of exceptional biological properties, including antifungal, antioxidant, antidiabetic, and anticancer properties [1,2]. Cur has been studied for its ability to alleviate cancer-related symptoms such as tiredness, neuropathic pain, and cognitive impairment. It also prevents metastasis and has anti-proliferative effects on cancer cells, including colon, prostate, and breast cancer [3]. In recent years, its anticancer and antioxidant properties have been widely used in clinical research to treat rheumatism, carcinogenesis, and oxidative stress-related pathology [4,5]. Cur medicine administered orally has been shown in phase I and phase II clinical studies to be very promising in individuals with colorectal neoplasia, pancreatic cancer, and breast cancer [6]. Cur has considerable therapeutic promise, but its clinical applications have been hampered by a variety of limitations, including poor water solubility and rapid metabolism [7]. Cur, consequently, has had its therapeutic potential reduced because of these limitations. As a result, nanocarriers have been developed to securely carry Cur to the desired locations.

The nanocarriers can be utilized for delivering chemotherapy drugs to specific locations, improving the efficacy of hazardous therapies. Engineered nanoparticles have a great deal of potential for improving diagnosis and therapy specificity. Nanoparticle development has evolved into a wide spectrum of clinical uses in recent years. To overcome the limits of free therapies and navigate biological barriers, polymeric/inorganic nanoparticles have been generated. However, the research into nanoparticles remains focused on improving delivery systems with a one-size-fits-all approach. As lipid-based, polymeric, and inorganic nanoparticles are made more precisely, they can be improved for drug administration [8]. To optimize the delivery of Cur, a variety of nanocarriers have been designed [9,10]. Cur encapsulation in biopolymeric particles, for example, or conjugation with nanoparticles, can be employed to increase Cur stability, absorption, and distribution to target areas. Polymer-based nanocarriers have been widely used to carry drugs because of their cytocompatibility, broad structural production and design capabilities, and intriguing physicochemical features [11,12,13].

We synthesized pH- and temperature-sensitive copolymeric nanogels in this study. Nanogels are nanoparticles formed by the combination of a hydrogel and a cross-linked hydrophilic polymer. Because they are easily made and customized, these nanogels have emerged as promising drug-carrying agents for targeting cancer cells. The temperature-sensitive polymer poly(N-isopropylacrylamide) (pNIPAm) has attracted the interest of researchers due to its abrupt phase transition feature. When the temperature changes, pNIPAm’s phase structure might shift from swelling to deswelling. Because of their reversible phase transition feature, pNIPAm polymers have been used to modify the drug loading and release process. Poly(allylamine) (pAm) is a water-soluble polymer with amine groups that is stable, non-resorbable, and biodegradable. Because of the large number of amine groups, it might be used to form drug-binding sites in drug delivery applications. Recently, it has been demonstrated that two or more polymers are frequently combined to produce a wide range of physicochemical properties. The pNIPAm was conjugated with the p(Am) to generate the p(NIPAm-co-Am) nanogels system to deliver the Cur. This synthetic approach is easy and more convenient compared to the multistep process of carious composites or nanomaterials. Furthermore, the proposed copolymer system is stimulated by the intracellular pH microenvironment and physiological body temperature. A variety of instrumental characterization techniques are used to characterize the synthesized p(NIPAm-co-Am) NG system, including ^1^H nuclear magnetic resonance (^1^H NMR) spectroscopy, Fourier transform infrared (FT-IR) spectroscopy, scanning electron microscopy (SEM), X-ray photoelectron spectroscopy (XPS), zeta potential, and particle size analysis. Furthermore, the in vitro drug delivery behavior of the p(NIPAm-co-Am) NG system was investigated under various pH (pH 7.2, 6.5, and 4.0) and temperature (25 °C, 37 °C, and 42 °C) conditions. In addition, MTT assay analysis was used to assess the biocompatibility of the p(NIPAm-co-Am) NG system on MDA-MB-231 cells. Overall, the findings of the experimental investigation show that the p(NIPAm-co-Am) NG system produced might be useful for dual pH and temperature-stimuli-responsive drug delivery applications.

## 2. Results and Discussion

### 2.1. Instrumental Characterizations

**Figure 1:**
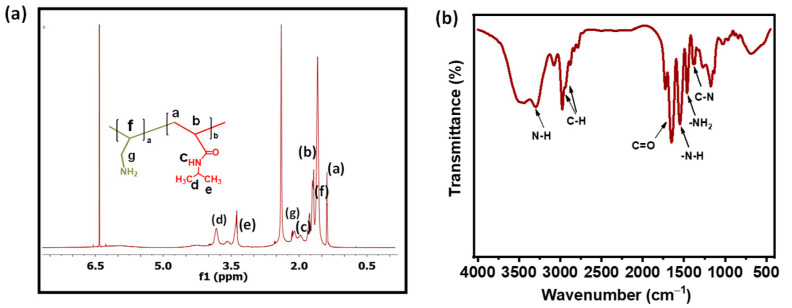
(**a**) ¹H NMR and (**b**) FT-IR spectra of p(NIPAm-co-Am) NG sample.

**Figure 2:**
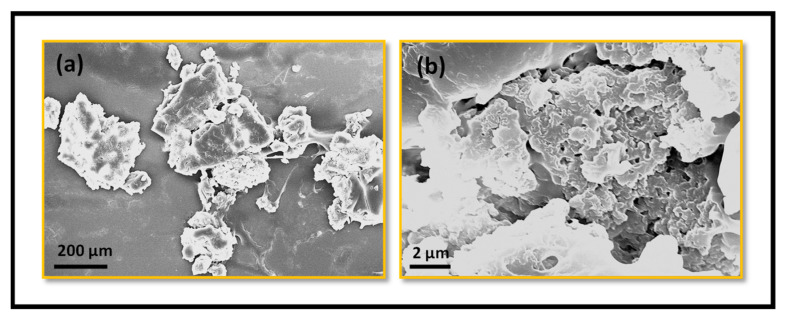
(**a**,**b**) SEM images represent the surface morphology of the p(NIPAm-co-Am) NG sample.

^1^H NMR and FT-IR measurements confirmed the successful incorporation of monomers such as NIPAm and Am in the p(NIPAm-co-Am) NG copolymer produced. Figure 1a shows a ^1^H NMR spectrum in which the proton resonance peak is at δ1.21 ppm and the resonance peak at δ3.79 ppm, indicating the methyl and amide groups of NIPAm monomer [14]. In addition, the resonance peak arose in the δ1.89–δ2.28 ppm region, confirming the existence of primary amine signals from the Am comonomer [15]. The ^1^H NMR spectra revealed the presence of NIPAm and Am in the p(NIPAm-co-Am) NG copolymer NG segments. Furthermore, the FT-IR spectroscopic analysis was performed to determine the structure of the p(NIPAm-co-Am) NG copolymer sample. As shown in Figure 1b, the characteristic vibration band at 1453 cm^−1^ indicates the stretching peak of C-N groups and the sharp peak at 1417 cm^−1^ indicates the N-H groups and the stretching mode of C=O groups of the NIPAm units present in the copolymer segments. In addition, the existence of an allylamine (Am) segment was confirmed by the characteristic peak appearing at 1452 cm^−1^, indicating the presence of primary amine (-N-H) groups. Moreover, the peaks appeared at 2972 cm^−1^ and 2926 cm^−1^, indicating alkyl carbon (C-H) stretching vibration bands. The bending vibration peak at 3293 cm^−1^ appeared for the -N-H bending mode of amine groups. A strong band appeared at 1544 cm^−1^ for the N-H stretching peak for allylamine groups. From the FT-IR spectral analysis, it could be confirmed that the allylamine units have been integrated into the p(NIPAm-co-Am) NG copolymer sample [16]. The morphological appearance of the developed p(NIPAm-co-Am) NG copolymer sample was examined using SEM analysis. Figure 2a,b shows aggregated irregular particles with rough surfaces in the dried sample. Aggregation may occur as a result of H-bonding interactions between the copolymer units’ C=O and N-H groups.

**Figure 3:**
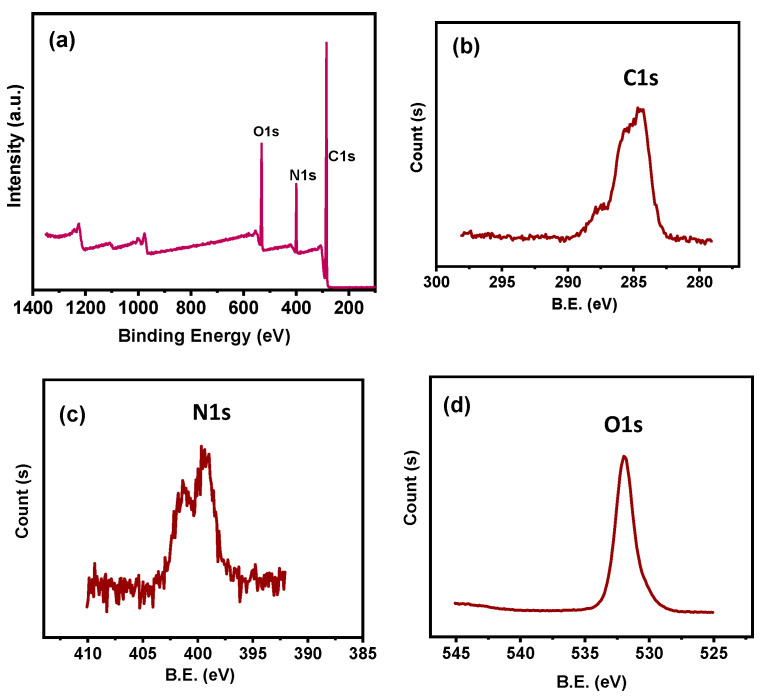
XPS spectra of (**a**) full scan spectrum; (**b**) C1s; (**c**) N1s; and (**d**) O1s elements, respectively.

The elemental composition of the resulting p(NIPAm-co-Am) NG copolymer sample was studied using XPS. The carbon, nitrogen, and oxygen signals were apparent in the broad scan spectra of the p(NIPAm-co-Am) NG copolymer sample (Figure 3a). The resonance peaks for carbon (C1s), nitrogen (N1s), and oxygen (O1s) components are at 284–290 eV, 396–402 eV, and 526–532 eV, respectively, indicating the existence of NIPAm and Am monomers in the copolymer segments (Figure 3) [17].

**Figure 4:**
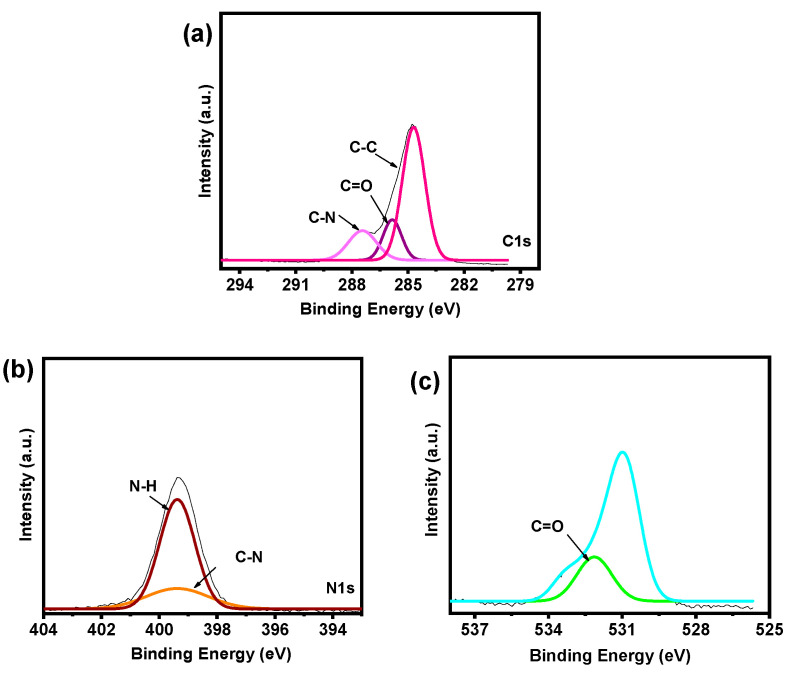
High-resolution XPS spectra of (**a**) C1s; (**b**) N1s; and (**c**) O1s elements, respectively.

In addition, Figure 4a–c shows a high-resolution spectrum of the binding energy of the various elements. The binding energy of the carbon spectrum for C1s was determined to be 284.7 eV, 286.5 eV, and 287.4 eV for C-C, C-H, and C-N binding, respectively (Figure 4a) [18]. The binding energy modes of nitrogen (N1s) were 398.1 eV and 399.4 eV for the C-N and N-H bonds of NIPAm and Am monomer segments, respectively (Figure 4b) [19]. Furthermore, the oxygen element (O1s) peak location for C=O groups of NIPAm units included in the p(NIPAM-co-Am) NG copolymer segments was 532.3 eV (Figure 4c) [20]. The existence of NIPAm and Am monomer segments in the p(NIPAM-co-Am) NG copolymer sample was verified using the XPS spectrum analysis findings.

### 2.2. Swelling–Deswelling Behavior and Phase Transition Mechanism

#### 2.2.1. pH-Responsive Behavior of the p(NIPAM-co-Am) NG

**Figure 5:**
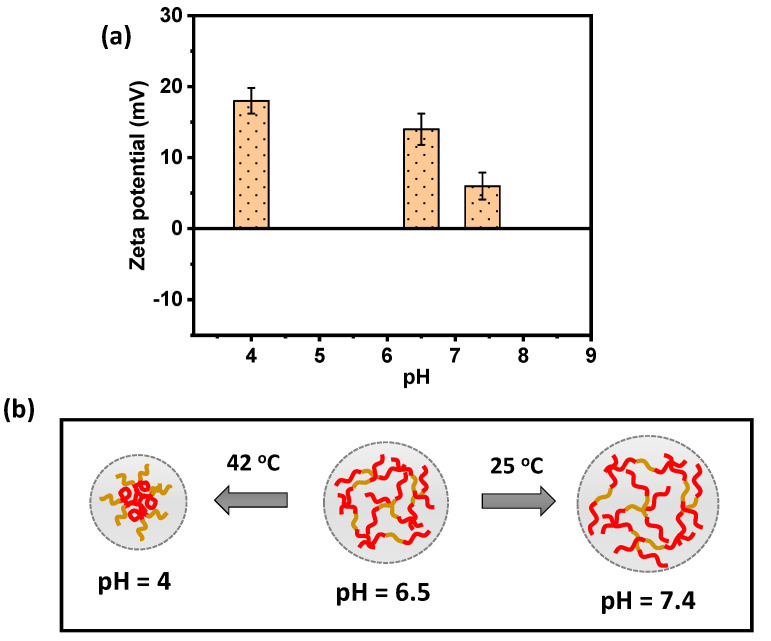
(**a**) Zeta potential of p(NIPAm-co-Am) NG system. (**b**) Schematic illustration of the phase transition of p(NIPAm-co-Am) NG under the pH and temperature stimuli conditions.

The amount of dielectric charge and the swelling–deswelling behavior of the synthesized p(NIPAm-co-Am) NG system under pH stimuli were assessed using zeta potential analysis. The p(NIPAm-co-Am) NG copolymer obtained exhibits pH responsiveness in the presence of primary amine (-NH_2_) groups in the polyallylamine segments. The amine-containing p(NIPAm-co-Am) NG system contains a cationic polyelectrolyte with a pKa value of around pKa 8–9 in aqueous solutions. As shown in Figure 5a, as the pH increased from 4.0 to 7.2, the zeta potential values decreased from +18 mV to +6 mV. The hydrophobic pNIPAm segments in the p(NIPAm-co-Am) NG aggregate into micelle cores due to the temperature-responsive phase transition from the linear structure to the coil-like globule structure, while the hydrophilic group packs outside the NG system, forming from the sol to gel phase transition. At higher pH levels, the p(NIPAm-co-Am) NG decreases protonation and increases hydrophilicity, allowing transition to the sol phase with liner copolymer, as illustrated in Figure 5b [21]. The temperature-induced phase transition behavior of the NIPAm segments and pH-induced protonation of the amine groups in the allylamine segments in the p(NIPAm-co-Am) NG copolymer system might explain this. Intramolecular hydrogen bonding takes place under basic pH conditions, but under acidic pH conditions, the amine groups of Am units are positively charged. The electrostatic repulsion between the protonated amino groups in the Am units disrupts intramolecular hydrogen bonding and causes the phase change from linear chain to globule structure.

#### 2.2.2. Temperature-Responsive Phase Transition Behavior of p(NIPAm-co-Am) NG System

**Figure 6:**
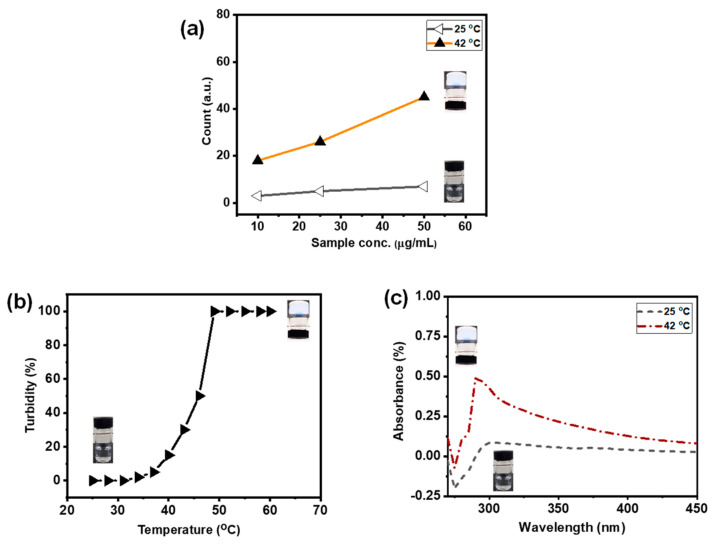
**Figure 6.** (**a**) DLS analysis of the p(NIPAm-co-Am) NG sample. (**b**) Relative turbidity of the p(NIPAm-co-Am) NG sample. (**c**) UV–vis absorption of the p(NIPAm-co-Am) NG sample at different solution temperatures.

The temperature-responsive sol–gel transition at 42 °C was verified using a dynamic light scattering (DLS) study. At 25 °C, the p(NIPAm) segments are linear and form H-bonds with the amine groups of the p(Am) segments. At higher temperatures (42 °C), the p(NIPAm) segments form globules and the p(Am) segments are exposed outside of the nanogels system, increasing the particle size of the p(NIPAm-co-Am) NG system. Figure 6a shows that at 25 °C, a constant DLS intensity was established for varying sample concentrations. The particle count, on the other hand, varied with the concentrations of the p(NIPAm-co-Am) NG sample at 42 °C. Furthermore, the p(NIPAm-co-Am) NG sample solution demonstrated a clear phase transition from a transparent solution at 25 °C to a turbid suspension at temperatures above the LCST (42 °C) (Figure 6a). The hydrophobic association serves as a cross-linking unit in the formation of stable nanogels [22].

The NG system’s relative turbidity was monitored from 25 °C to 60 °C, with an equilibrium period of 5 min at each temperature (Figure 6b). The p(NIPAm-co-Am) NG sample dispersion was homogenous and transparent at <40 °C, with a transmittance of about 100%. Because of the high transmittance, the p(NIPAm-co-Am) NG copolymer chains were significantly swelled and exited at the linear structure [23,24]. There was a substantial decrease in transmittance starting at 40 °C. Above 40 °C, the relative turbidity of the samples increases to 100%, suggesting that the p(NIPAm-co-Am) NG sample was in the gel phase. The UV–vis absorption of the p(NIPAm-co-Am) NG sample was also tested at 25 °C and 42 °C (Figure 6b). As shown in Figure 6c, the p(NIPAm-co-Am) NG solution had a relatively low absorption at 25 °C, which might be attributed to the presence of a transparent and linear polymer structure at that temperature. However, at temperatures over 42 °C, substantial UV–vis absorption was found, which might be related to temperature-induced micelle generation, which induces solution turbidity [25].

### 2.3. Discussion about Swelling–Deswelling Behavior and Phase Transition Mechanism

The copolymer hydrogel system’s dual pH and temperature responsiveness is regarded as a significant feature [26]. The physical stimulus must undergo a change in phase rather than a chemical stimulus. At room temperature, the p(NIPAm-co-Am) NG sample may be dissolved in water to generate a non-crosslinked homogenous solution. The hydrophobic block of pNIPAm facilitates gelation of the p(NIPAm-co-Am) NG copolymer sample. When a suitable hydrophobic–hydrophilic balance is maintained, an amphiphilic copolymer forms hydrophobic domains surrounded by hydrophilic polymer segments [27].

**Figure 7:**
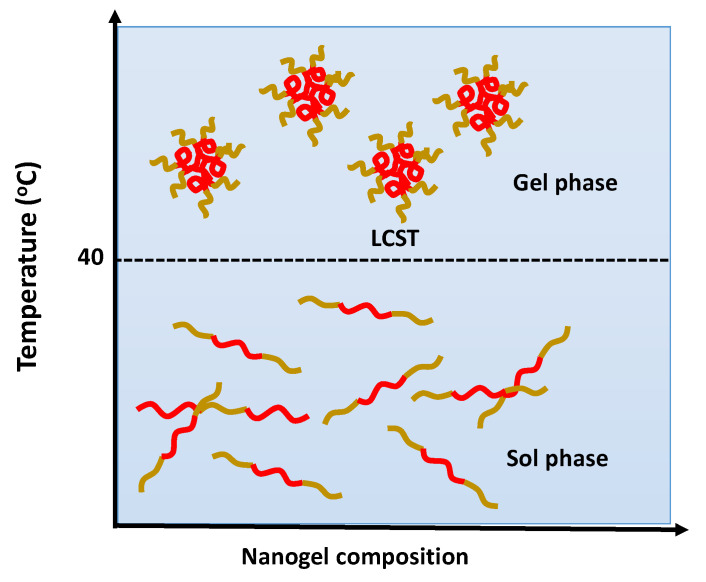
A proposed schematic illustration of the p(NIPAm-co-Am) NG system conformational change in each phase state.

The NIPAm segments in the p(NIPAm-co-Am) NG system is composed of both hydrophilic amide (-CO-NH-) groups and hydrophobic isopropyl (-CH(CH_3_)_2_) side chains and have a lower critical solution temperature (LCST) of 40 °C. In an aqueous solution, the pNIPAm undergoes phase change at about 32 °C [28]. When the NIPAm has copolymerized with the Am comonomer, the resulting p(NIPAm-co-Am) NG sample has an LCST greater than 40 °C [29]. Therefore, when the solution temperature rose beyond 40 °C, the p(NIPAm-co-Am) NG sample demonstrated a distinct phase change from sol to gel (Figure 7). Because the LCST of the p(NIPAm-co-Am) NG sample is higher than that of the body, quick gelation of these nanogels can be avoided when the samples are injected into the body. Maintaining fine temperature control allows for site-specific drug delivery and controlled drug release. This result is a well-organized structure in the solution that can transport and encapsulate the drugs. Cur molecules can be easily loaded into the p(NIPAm-co-Am) NG sample by first dissolving the Cur in the solution at a lower temperature and then raising the temperature to above the LCST for gelation. The low temperature is used to mix copolymers and Cur and protects the Cur from denaturation or aggregation.

### 2.4. Stimuli-Responsive Drug Delivery Behavior of the p(NIPAm-co-Am) NG System

**Scheme 1:**
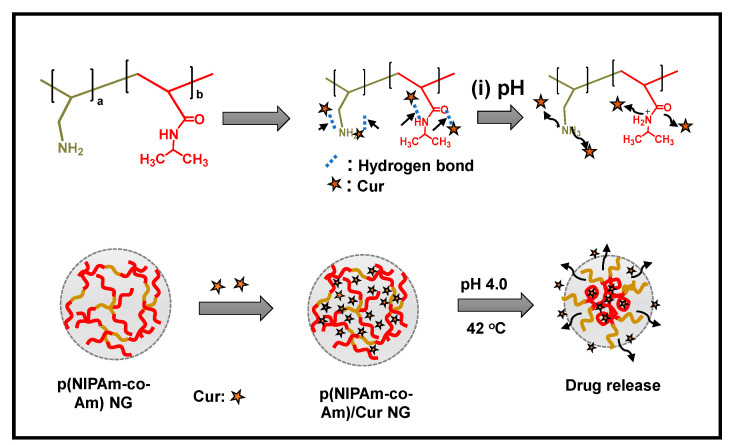
Schematic representation of drug loading and stimuli-responsive release behavior of the p(NIPAm-co-Am) NG system.

Ionic interactions produce the pH-based phase change in the p(NIPAm-co-Am) NG sample (Scheme 1). Protons (H^+^ ions) are attracted to the amine, amide, and carbonyl groups of the p(NIPAm-co-Am) NG sample [30]. When the p(NIPAm-co-Am) NG sample is placed in an aqueous solution, the existing functional groups crosslink with water [31]. At low pH, the p(NIPAm-co-Am) NG system undergoes protonation, allowing the drug to be delivered into the tumor’s low pH microenvironment.

#### 2.4.1. In Vitro Cur Delivery Behavior of the p(NIPAm-co-Am) NG/Cur System

The pH and temperature-responsive drug release characteristics of the Cur loaded p(NIPAm-co-Am) NG/Cur system were investigated at different pH (pH 7.2, 6.5, and 4.0); different temperatures (25 °C, 37 °C, and 42 °C); and at the combined pH and temperature (pH 7.2/42 °C and pH 4.0/42 °C), respectively, using a bioactive agent Cur as a model drug.

**Figure 8:**
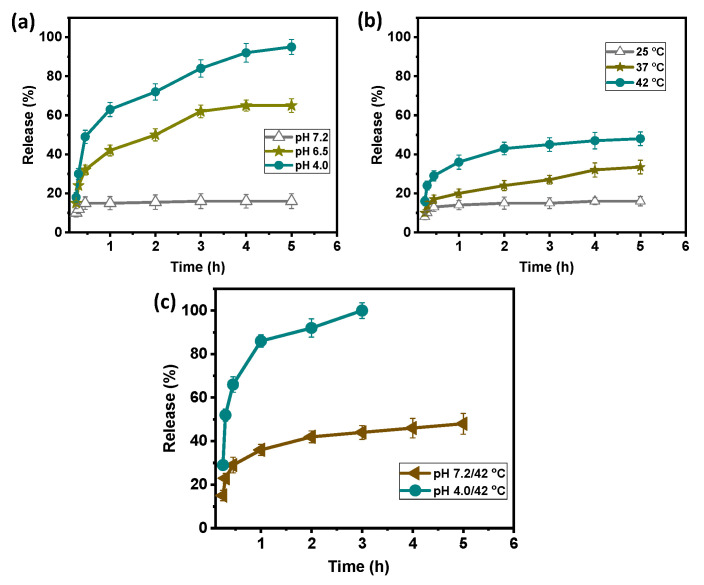
In vitro Cur delivery of p(NIPAm-co-Am) NG. (**a**) Cur release under pH stimuli, (**b**) Cur release under temperature stimuli, and (**c**) Cur release under pH and temperature stimuli conditions, respectively.

Firstly, the Cur release behavior of the Cur loaded p(NIPAm-co-Am) NG/Cur system was examined for 5 h at 25 °C under varied circumstances (pH 7.2, 6.5, and 4.0). The pH-responsive release profile of the p(NIPAm-co-Am) NG/Cur system is shown in Figure 8a. As shown in Figure 8a, approximately ~16% of the Cur was released in 5 h at pH 7.2. In comparison, approximately ~63% and >90% of the Cur release occurred within 5 h at pH 6.5 and pH 4.0, respectively. The increased Cur release at acidic pH (pH 4.0) than at physiological pH (pH 7.2) could be due to the protonation of drug binding sites such as amine (-NH_2_) and amide (CO-NH-) groups of Am and NIPAm groups, which causes electrostatic repulsion between the protonated drug binding functional groups and the Cur molecules [32]. Secondly, the Cur release behavior of the p(NIPAm-co-Am) NG/Cur system was examined under several temperature-stimuli settings, such as 25 °C, 37 °C, and 42 °C, while keeping the solution pH at around pH 7.2. As demonstrated in Figure 8b, the Cur release was rather low, with only approximately ~16% released in 5 h at pH 25 °C. Cur release, on the other hand, was noticeably increased to about ~33% and ~45% at 37 °C and 42 °C in 5 h, which could be due to the temperature-induced phase transition of NIPAm segments in the p(NIPAm-co-Am) NG/Cur system, and, thus, the physisorbed Cur molecules were released out of the p(NIPAm-co-Am) NG/Cur system.

Finally, the p(NIPAm-co-Am) NG/Cur system’s combination pH and temperature-responsive Cur release behavior were investigated. At pH 7.2/42 °C, the p(NIPAm-co-Am) NG/Cur system released approximately ~45% of the Cur, as illustrated in Figure 8c. In contrast, a considerable increase in Cur release was found at pH 4.0/42 °C, with almost ~100% of Cur being released in 3 h.

Compared to the only pH (pH 4.0, ~90%) or only temperature (42 °C, ~45%) conditions, the combined stimuli (pH 4.0/42 °C) condition showed an enhanced and almost complete Cur release in 3 h, which might be due to the dual effects such as pH-stimuli induced protonation and temperature-induced phase transition (linear chain to globular structure) which combine to push out the Cur molecules from the p(NIPAm-co-Am) NG/Cur system (Scheme 1).

The experimental results indicated that the combination of dual stimuli such as pH and temperature resulted in enhanced drug release efficiency of the synthesized p(NIPAm-co-Am) NG system. In comparison to the NIPAm-based thermoresponsive copolymer-based drug delivery system reported in the literature (Table 1) [33,34,35,36,37,38,39], which focused on thermoresponsive behavior, the proposed p(NIPAm-co-Am) NG/Cur system demonstrated efficacy for dual pH and temperature-stimuli-responsive and controlled drug delivery applications.

#### 2.4.2. In Vitro Cytocompatibility Study

**Figure 9:**
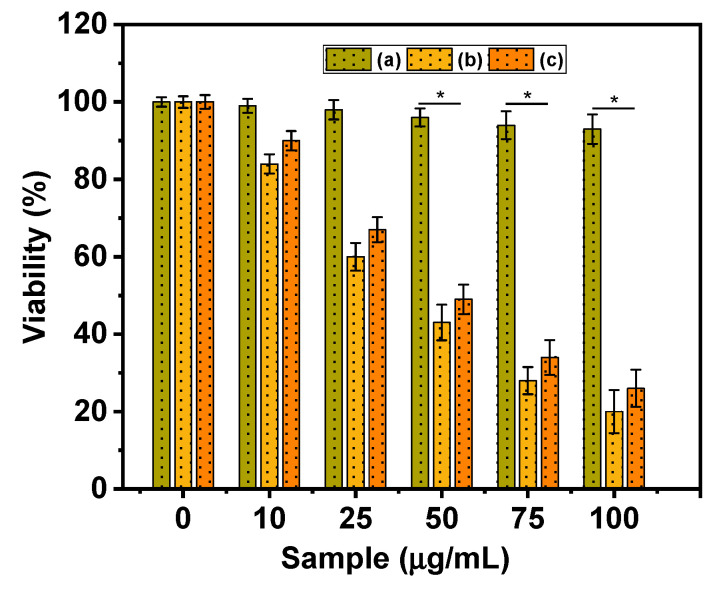
In vitro biocompatibility of (a) p(NIPAm-co-Am) NG; (b) Cur loaded p(NIPAm co-Am) NG/Cur; and (c) pure Cur, respectively, at 37 °C. Statistical significance to the cell toxicity with different samples (*, significant *p* < 0.05).

An MTT assay study was performed on MDA-MB-231 cells to determine the in vitro cytocompatibility of synthesized p(NIPAm-co-Am) NG, Cur loaded p(NIPAm-co-Am) NG/Cur, and free Cur at 37 °C. As shown in Figure 9, the p(NIPAm-co-Am) NG without Cur loading demonstrated ~92% cell viability in MDA-MB-231 cells. The Cur loaded p(NIPAm-co-Am) NG/Cur sample, on the other hand, demonstrated considerable cell toxicity with respect to sample concentrations. As shown in Figure 9, the p(NIPAm-co-Am) NG/Cur sample was more toxic to MDA-MB-231 cells than pure Cur at equivalent dosages. This might be because the gradual release of Cur from the p(NIPAm-co-Am) NG/Cur sample induced more cell toxicity than pure Cur alone [40,41]. Because of the MTT assay results, the synthesized p(NIPAm-co-Am) NG system is biocompatible and might be used for pH and temperature-responsive drug delivery applications.

## 3. Conclusions

In conclusion, a thermoresponsive p(NIPAm-co-Am) NG copolymer system based on NIPAm and Am monomers was developed for dual pH and temperature-responsive drug delivery applications. Various instrumental analytical techniques were used to examine the developed copolymer. The p(NIPAm-co-Am) NG copolymer shows aggregated irregular particles with rough surfaces. The zeta potential values showed about +18 mV to +6 mV in the pH range from pH 4 to pH 7.2. In addition, the p(NIPAm-co-Am) NG system showed a significant temperature-induced sol–gel phase transition brought about by the behavior and temperature-induced protonation of amine groups. Cur, a bioactive substance, was utilized to investigate the loading and pH and temperature-responsive release behavior of the p(NIPAm-co-Am) NG system at various pH levels (pH 7.2, 6.5, and 4.0) and temperatures (25 °C, 37 °C, and 42 °C) stimuli conditions, respectively. The Cur loading into the p(NIPAM-co-Am) NG/Cur was estimated to be about ~30.2%. The combined (pH and temperature) stimuli improved the drug release properties of the Cur-loaded p(NIPAm-co-Am) NG/Cur system over the pH (4.0) or temperature (42 °C) stimuli alone. Furthermore, the in vitro cytocompatibility evaluation study results indicate that the synthesized p(NIPAm-co-Am) NG system is biocompatible at the sample concentrations tested (0–100 µg/mL). Overall, the experimental study results supported the conclusion that the p(NIPAm-co-Am) NG system could be applicable and could be utilized for pH and temperature-responsive controlled drug delivery applications.

## 4. Experimental and Characterizations

### 4.1. Chemicals and Reagents

N-Isopropylacrylamide (NIPAm, 97%), allylamine (Am, 98%), 2,2-azoisobutyronitrile (AIBN), curcumin (Cur), tetrahydrofuran (THF), hexane, and toluene were purchased from Sigma Aldrich Chemical Co., Saint Louis, MO, USA, and used as received.

### 4.2. Synthesis of p(NIPAm-co-Am) Copolymer NG System

**Scheme 2:**
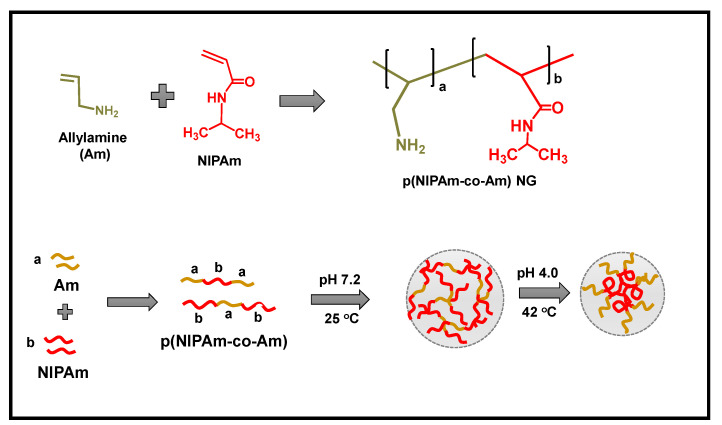
Illustration of the (a) synthesis of p(NIPAm-co-Am) copolymer NG system and (b) stimuli-responsive phase transition of p(NIPAm-co-Am) NG sample.

Approximately 2.0 g (17.6 mmol) of NIPAm monomer was dissolved in a 100 mL round-bottomed flask containing 25 mL of THF solvent to complete this synthesis. Because both the NIPAm and Am monomers are readily soluble in THF, we chose THF as an appropriate solvent for this polymerization process. To this, about 1.0 g (4.09 mmol) of Am monomer was then added and the reaction flask was degassed for 30 min by purging it with nitrogen gas. Then, around 0.1 g of AIBN initiator was added and the polymerization reaction was allowed to continue for 24 h at 68 °C in an inert environment. After the polymerization process was completed, the resulting viscous mass was precipitated in hexane (250 mL). The precipitation procedure was repeated three times before being rinsed with hexane and dried overnight under vacuum conditions. The resulting copolymer was designated as p(NIPAm-co-Am) NG (Scheme 2).

### 4.3. Instrumental Characterizations

^1^H NMR was used to analyze the chemical structure of the synthesized p(NIPAm-co-Am) NG system using NMR instrument (OXFORD, 600 mHz). The morphology of the synthesized p(NIPAm-co-Am) NG system was observed using SEM analysis (JEOL 6400 instrument) at 10 kV accelerating voltage. XPS (XPS, Tucson, AZ, USA) analysis was performed to verify the chemical composition of the samples. FT-IR spectroscopy (JASCO FT-IR 4100 spectrometer) was used to analyze the sample using the KBr pelleting method. Zeta potential and particle size analysis were performed on a Malvern Zetasizer (Nano ZS, Malvern, UK). UV–vis absorption spectra were recorded using a UV–vis spectrometer (Cary 60, Agilent Inc., Santa Clarita, CA, USA).

### 4.4. Cur Loading into the p(NIPAm-co-Am) NG System

Cur was employed as a model bioactive agent to assess the drug loading and stimuli-responsive release behavior of the synthesized p(NIPAm-co-Am) NG system under various pH and temperature stimulus conditions. To load Cur molecules, the hydrogel swelling method was used. Approximately 0.1 g of p(NIPAm-co-Am) NG sample was dissolved in 15 mL deionized water to perform the Cur loading. To this, 1 mL of Cur solution (50 mg/mL in ethanol) was added and swirled for 12 h in the dark. Following loading, the Cur-encapsulated sample was precipitated and dried at room temperature under vacuum conditions. The UV–vis spectrophotometric approach was used to determine the Cur loading into the developed p(NIPAm-co-Am) NG system. The Cur loading was calculated as follows: Cur loading capacity (%) = (Wt. of the entrapped Cur inside the gel/Total Wt. of the gel) × 100. The Cur loaded sample was named p(NIPAm-co-Am) NG/Cur. The Cur loading into the p(NIPAM-co-Am) NG/Cur was estimated to be about ~30.2%.

### 4.5. In Vitro Cur Release Study

The in vitro Cur release behavior of the p(NIPAm-co-Am) NG system was investigated using various release study conditions, including (i) different pH (pH 7.2, 6.5, and 4.0); (ii) different temperatures (25 °C, 37 °C, and 42 °C); and the combined pH and temperature (pH 7.2/42 °C and 4.0/42 °C), respectively. In this investigation, 50 mg of Cur-loaded p(NIPAm-co-Am) NG/Cur samples were placed in a dialysis membrane bag. The sample-impregnated membrane bag was put in a 25 mL beaker containing 10 mL of phosphate buffer saline (PBS) solution and stored in the dark. The pH of the medium was kept constant by employing an acidic buffer, and the temperature of the release media was kept constant by utilizing a water bath coupled with a magnetic stirrer. At predefined time intervals, about 1 mL of the release medium was taken out, and the amount of released Cur was measured using a UV–vis spectrophotometer set to 427 nm. The Cur calibration curve was used to calculate the amount of released Cur from the Cur loaded p(NIPAm-co-Am) NG/Cur samples. The released Cur was determined as follows: Cur release (%) = (Cur release at time t/Amount of sample taken) × 100.

### 4.6. In Vitro Cytocompatibility Study

The in vitro cytocompatibility of the synthesized p(NIPAm-co-Am) NG sample, Cur loaded p(NIPAm-co-Am) NG/Cur sample, and free Cur was investigated on MDA-MB-231 cells at various sample concentrations (0–100 µg/mL). MDA-MB-231 cells (1 × 10^4^ cells/well) were seeded onto 96-well plates and cultivated at 37 °C for 24 h. After 24 h, the existing media were replaced with fresh media containing different amounts of samples and cultured for another 24 h. After removing the media, a fresh MTT solution was added to each well and incubated for 4 h. After 4 h of incubation, the formazan crystals were solubilized with 20 µL of DMSO solution. The absorbance of each well was measured at 595 nm using a microplate reader (EL800, BioTek Instruments, Winooski, VT, USA).

### 4.7. Statistical Analysis

All results, expressed as the mean ± SD, were analyzed using a two-tailed Student’s *t*-test or one-way analysis of variance (ANOVA). The acceptable level of significance was *p* < 0.05.

## Figures and Tables

**Table 1:** Various types of pNIPAm-based copolymers for drug delivery.

Polymers	Stimuli for Drug Delivery	References
Biodegradable thermoresponsive polymers	Temperature	[33]
Thermosensitive Polymers	Temperature	[34]
NIPAM-acrylamide-methacrolein copolymer	Temperature	[35]
Magnetic NPs-PNIPAM copolymer	Temperature	[36]
Albumin and poly(allylamine)	Temperature	[37]
Thermoresponsive gating particles	Temperature	[38]
PNIPAM-coated Au@Mesoporous-SiO_2_ NPs	Temperature	[39]
p(NIPAm-co-Am) NG	pH and Temperature	This work

## Data Availability

Not applicable.
